# Gut Microbiota and Tacrolimus Dosing in Kidney Transplantation

**DOI:** 10.1371/journal.pone.0122399

**Published:** 2015-03-27

**Authors:** John R. Lee, Thangamani Muthukumar, Darshana Dadhania, Ying Taur, Robert R. Jenq, Nora C. Toussaint, Lilan Ling, Eric Pamer, Manikkam Suthanthiran

**Affiliations:** 1 Department of Medicine, Division of Nephrology and Hypertension, Weill Cornell Medical College, New York, New York, United States of America; 2 Department of Transplantation Medicine, New York Presbyterian Hospital—Weill Cornell Medical Center, New York, New York, United States of America; 3 Department of Medicine, Infectious Diseases Services, Memorial Sloan Kettering Cancer Center, New York, New York, United States of America; 4 Lucille Castori Center for Microbes, Inflammation and Cancer, Memorial Sloan Kettering Cancer Center, New York, New York, United States of America; 5 Weill Cornell Medical College, New York, New York, United States of America; 6 Department of Medicine, Adult Bone Marrow Transplant Service, Memorial Sloan Kettering Cancer Center, New York, New York, United States of America; University of Toledo, UNITED STATES

## Abstract

Tacrolimus dosing to establish therapeutic levels in recipients of organ transplants is a challenging task because of much interpatient and intrapatient variability in drug absorption, metabolism, and disposition. In view of the reported impact of gut microbial species on drug metabolism, we investigated the relationship between the gut microbiota and tacrolimus dosing requirements in this pilot study of adult kidney transplant recipients. Serial fecal specimens were collected during the first month of transplantation from 19 kidney transplant recipients who either required a 50% increase from initial tacrolimus dosing during the first month of transplantation (Dose Escalation Group, n=5) or did not require such an increase (Dose Stable Group, n=14). We characterized bacterial composition in the fecal specimens by deep sequencing of the PCR amplified 16S rRNA V4-V5 region and we investigated the hypothesis that gut microbial composition is associated with tacrolimus dosing requirements. Initial tacrolimus dosing was similar in the Dose Escalation Group and in the Stable Group (4.2±1.1 mg/day vs. 3.8±0.8 mg/day, respectively, P=0.61, two-way between-group ANOVA using contrasts) but became higher in the Dose Escalation Group than in the Dose Stable Group by the end of the first transplantation month (9.6±2.4 mg/day vs. 3.3±1.5 mg/day, respectively, P<0.001). Our systematic characterization of the gut microbial composition identified that fecal *Faecalibacterium prausnitzii* abundance in the first week of transplantation was 11.8% in the Dose Escalation Group and 0.8% in the Dose Stable Group (P=0.002, Wilcoxon Rank Sum test, P<0.05 after Benjamini-Hochberg correction for multiple hypotheses). Fecal *Faecalibacterium prausnitzii* abundance in the first week of transplantation was positively correlated with future tacrolimus dosing at 1 month (R=0.57, P=0.01) and had a coefficient±standard error of 1.0±0.6 (P=0.08) after multivariable linear regression. Our novel observations may help further explain inter-individual differences in tacrolimus dosing to achieve therapeutic levels.

## Introduction

The clinical introduction of calcineurin inhibitors has significantly reduced acute rejection rates and increased graft survival in kidney transplant recipients [1]. In 2012, over 90% of kidney transplant recipients were maintained on calcineurin inhibitors with the majority being treated with tacrolimus [2]. Tacrolimus, however, possesses a narrow therapeutic index with sub-therapeutic levels leading to immune rejection and supra-therapeutic levels leading to nephrotoxicity and neurotoxicity [3]. Complicating the narrow therapeutic index is the difficulty in predicting initial tacrolimus dosing to maintain therapeutic trough levels.

Clinical and genetic characteristics associated with tacrolimus pharmacokinetics such as age, gender, race, corticosteroid therapy, hemoglobin concentration, albumin concentration, drug interactions, liver function, and CYP3A5 polymorphisms have been reported [4–11]. Nevertheless, these factors do not completely account for the inter-individual variability in tacrolimus dosing required to achieve therapeutic trough levels in individual patients [12].

Recently, the gut microbiota has been implicated in the metabolism of drugs with specific microbial species being associated with direct and indirect drug metabolism [13–16]. While the gut dysbiosis has not been directly associated with tacrolimus metabolism, several lines of evidence support this possibility. For example, post-transplant diarrhea and enterocolitis have been associated with altered tacrolimus trough levels [17–20]. Although down-regulation of CYP3A4 and P-glycoprotein in the intestinal epithelial cells has been advanced as a potential mechanism [18, 21], the biologic mechanisms for the alterations in tacrolimus drug levels in patients with post-transplant gastrointestinal complications have not been elucidated. Also, antibiotics such as quinolones, cephalosporins, and metronidazole, which all have the potential to affect the gut microbiome, have been associated with altered tacrolimus trough levels [22–24].

We have recently reported on the relationship of the gut microbiota and kidney transplantation outcomes such as post-transplant diarrhea, *Enterococcus* urinary tract infections, and acute rejection [25]. In the current investigation, we studied a subset of kidney transplant recipients from our earlier study who all had available fecal specimens during the first week of transplantation, who were continued on tacrolimus maintenance immunosuppression, and who did not develop acute rejection in the first month of transplantation (n = 19). We characterized the fecal microbiota in these 19 kidney transplant recipients during the first month of kidney transplantation, a critical period in which tacrolimus is adjusted to achieve therapeutic drug levels. Our systematic characterization of the fecal microbiota by 16S rRNA deep sequencing identified that the abundance of fecal *Faecalibacterium prausnitzii* early after kidney transplantation is associated with tacrolimus dosing requirements in kidney transplant recipients.

## Materials and Methods

### Study cohort

Between August 2012 and January 2013, 19 kidney transplant recipients provided fecal specimens during the first week following transplantation and approximately every two weeks thereafter. The subjects provided the fecal specimens within one day of production and the specimens were frozen at -80°C. The Institutional Review Board at Weill Cornell Medical College approved the study protocol and the subjects provided informed written consent.

### Kidney transplant protocol

All 19 kidney transplant recipients received either anti-thymocyte globulin or basiliximab as induction therapy. Maintenance oral immunosuppressive therapy consisted of a combination regimen of tacrolimus and mycophenolate with or without maintenance prednisone. A single dose of antibiotics was administered prior to surgery, and consisted primarily of cefazolin and vancomycin in cases of penicillin allergy. Transplant recipients also received valgancyclovir or acyclovir for 6 months for cytomegalovirus prophylaxis, clotrimazole twice daily for 3 months for thrush prophylaxis, and trimethoprim/sulfamethoxazole for 12 months for *Pneumocystis jiroveci* prophylaxis. In those with sulfa allergy, either dapsone or atovaquone was used. Clinical and demographical characteristics were also obtained regarding the 19 subjects from electronic medical records.

### 16S rRNA V4–V5 PCR amplification

DNA was isolated from each fecal specimen using a phenol-chloroform / bead beater disruption isolation method as previously described [26]. The V4–V5 variable region of the 16S rRNA encoding gene was amplified using PCR assay. Specifically, duplicate 50-μL PCR reactions were established containing 50 ng of purified DNA, 1.5 mM MgCl2, 2.5μL of 10X PCR buffer, 0.2 mM dNTPs, 1.25 U Platinum Taq DNA polymerase, and 0.2 μM of forward primer (563F [5’-nnnnnnnn-NNNNNNNNNNNN-AYTGGGYDTAAAGNG-3’] and reverse primer (926R [5’-nnnnnnnn-NNNNNNNNNNNN-CCGTCAATTYHTTTRAGT-3’]) designed to cover the V4–V5 hypervariable region. The primers contained a unique 12 base Golay barcode for sample identification and 1–8 additional nucleotides preceding the barcode to offset the sequencing of the primers [27]. The PCR cycling conditions included: 94°C for 3 minutes, followed by 27 cycles of 94°C for 50 seconds, 51°C for 30 seconds, and 72°C for 1 minute with 72°C for 5 min as the elongation step. Duplicate PCR products were pooled and then purified using the Qiagen PCR purification kit. Further details on the 16S rRNA V4–V5 PCR amplification can be found in [25].

### 16S rRNA deep sequencing and sequencing analysis

PCR products were quantified using the Agilent Bioanalyzer and Illumina barcodes and adaptors were ligated, utilizing the Illumina TruSeq Sample Preparation kit. The PCR products were then sequenced using an Illumina Miseq instrument (250 by 250 base pair).

Read pairs were merged using mothur version 1.31.1 [28]. Sequences longer than 400 base pairs, sequences containing homopolymer stretches longer than 8 base pairs or containing undetermined bases, sequences with no exact match to the primer with up to 3 mismatches or that did not align to the V4–V5 16S rRNA variable region were not included in further analysis. Alignment to the V4–V5 region (SILVA reference) [29] was performed using mothur (Needleman-Wunsch algorithm); chimeric sequences were removed using Uchime [30]. Pre.cluster in mothur was utilized to reduce the effects of sequencing errors in overestimating microbial diversity [31]. Sequences were grouped into operational taxonomic units using the average neighbor algorithm and grouped on the basis of 97 percent or greater similarity. Phylogenetic classification was performed with the Bayesian classifier algorithm using a bootstrap cutoff of 60% [32]. Further details on the 16S rRNA deep sequencing and sequencing analysis can be found in [25].

### Tacrolimus grouping

Per our transplant center standard immunosuppression protocol, kidney transplant recipients were initially prescribed 4 mg/day of tacrolimus by the oral route in two divided doses unless clinically relevant drug interactions were present. Tacrolimus dosing in each patient was then adjusted to achieve a target therapeutic level of 8 to 10 ng/mL during the first month of transplantation. Tacrolimus trough levels were measured approximately twice a week for the first month and tacrolimus dosages were adjusted based on tacrolimus trough levels. All tacrolimus levels were measured at New York Presbyterian Hospital—Weill Cornell Medical Center’s clinical laboratory using liquid chromatography tandem mass spectrometry (TQD System, Waters, Milford, MA).

Based on the tacrolimus dosing at day 28 post-transplantation, we characterized the subjects into two groups: Dose Escalation Group (subjects requiring a 50% increase from standard initial dosing to attain target drug levels of 8 to 10 ng/mL) (tacrolimus dosing > 6 mg/day) and Dose Stable Group (subjects not requiring a 50% dose escalation) (tacrolimus dosing ≤ 6 mg/day). We evaluated whether this differential tacrolimus dosing requirement was related to gut microbial composition.

### Statistical analyses

Shannon diversity index for each sample was measured using mothur [28] and a locally estimated scatterplot smoothed (LOESS) curve was constructed for evaluating the sequentially collected fecal specimens. A phylogenetic tree was constructed based on the 16S sequence alignment using clearcut in mothur [28, 33]. Unweighted UniFrac was run using this tree and principal coordinate analysis (PCoA) was performed on the resulting distance matrix [34]. To compare the most common taxons in the first week fecal specimens between the Dose Escalation Group and the Dose Stable Group, we utilized Wilcoxon rank sum tests and the Benjamini-Hochberg correction for multiple hypotheses at each taxonomic level. Fisher’s exact test was used for group comparisons involving dichotomous variables and continuous variables were compared using Wilcoxon rank sum tests (for unpaired groups) and Wilcoxon sign rank tests (for paired groups). Values that were repeatedly measured in two groups over time were compared using a two-way between-group ANOVA using contrasts to compare the two groups at each time point. Correlations between 2 continuous variables were evaluated using a Pearson’s correlation and a univariate linear regression (for log transformed values, a 0 value was assigned half the lowest value in the series). Variables that had a significant linear correlation with tacrolimus dosing (P<0.10) were computed in a multivariable linear regression model. All calculations were performed using STATA 12.1 I/C and R 3.1.1.

## Results

### Study cohort characteristics

Per our standard transplant protocol, tacrolimus dosing was titrated in the 19 kidney transplant recipients to achieve tacrolimus trough levels of 8 to 10 ng/mL in the first month of transplantation. All 19 kidney transplant recipients received clotrimazole twice daily as anti-fungal prophylaxis during the first month of transplantation. Two of the 19 recipients were treated with diltiazem for clinical reasons. No other patient received CYP3A4 inhibitors during the first month of transplantation. 3 patients reported antibiotic use (one subject received amoxicillin, another received amoxicillin, and another received clindamycin) within one month prior to transplantation.

### Baseline characteristics of the tacrolimus dosing groups

Among the 19 kidney transplant recipients, 5 patients required a 50% increase from standard initial tacrolimus dosing by 1 month post-transplantation (Dose Escalation Group) (tacrolimus dosing > 6 mg/day) and the remaining 14 patients did not require a 50% increase from initial standard tacrolimus dosing (Dose Stable Group) (tacrolimus dosing ≤ 6 mg/day) during the same period. The clinical and transplant characteristics of the study groups are summarized in Table 1. As shown, variables that could potentially affect tacrolimus dosing such as age, weight, gender, race, type of transplantation (living donor vs. deceased donor), and steroid maintenance therapy were not significantly different between the two groups. As shown in Table 1, the type of induction therapy and the infection prophylaxis therapies were also not significantly different between the two groups.

**Table 1:** Clinical Characteristics of the Transplant Cohort.

Characteristics	Dose Escalation	Dose Stable	P value^a^
	Group (N = 5)	Group (N = 14)	
Age (Mean±SD)	43.4±19.0	58.8±7.8	0.16
Weight (Mean±SD)	81.2±13.6	74.1±14.1	0.23
Male Gender	4 (80.0%)	4 (28.6%)	0.11
African American Race	0 (0.0%)	3 (21.4%)	0.53
Deceased Donor Transplantation	2 (40.0%)	6 (42.9%)	0.99
Induction Therapy			
Anti-thymocyte globulin	5 (100.0%)	11 (78.6%)	0.53
Basiliximab	0 (0.0%)	3 (21.4%)	0.53
Maintenance Therapy			
Tacrolimus and Mycophenolate	5 (100.0%)	14 (100.0%)	0.99
Steroid Maintenance Protocol	1 (20.0%)	6 (42.9%)	0.60
Prophylaxis Therapy			
Preoperative Antibiotic Prophylaxis			
Cefazolin	5 (100.0%)	11 (78.6%)	0.53
Vancomycin	0 (0.0%)	3 (21.4%)	0.53
Clotrimazole	5 (100.0%)	14 (100.0%)	0.99
CMV Prophylaxis			
Valgancyclovir	4 (80.0%)	13 (92.9%)	0.47
Acyclovir	1 (20.0%)	1 (7.1%)	0.47
PCP Prophylaxis			
Trimethoprim/Sulfamethoxazole	5 (100.0%)	11 (78.6%)	0.53
Dapsone or Atovaquone	0 (0.0%)	3 (21.4%)	0.53

^a^ Categorical variables were compared using Fisher’s exact test and continuous variables were compared using the Wilcoxon Rank-Sum test.

**Fig 1:**
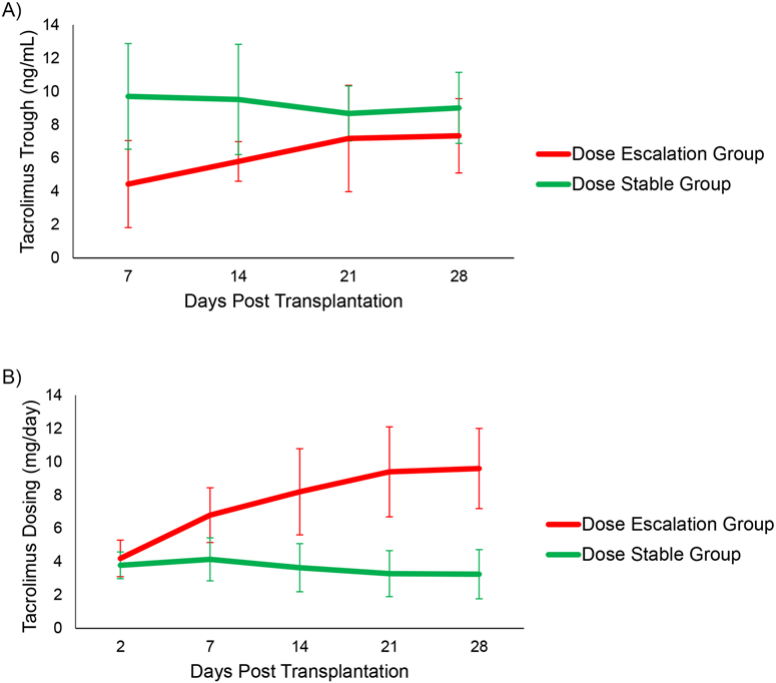
Tacrolimus Trough Levels and Tacrolimus Dosing in the Kidney Transplant Cohort. A) Tacrolimus troughs are shown over the course of the first month. On the x-axis is the day after kidney transplantation and on the y-axis is the mean tacrolimus trough of the group (ng/mL) corresponding to the day on the x-axis. The Dose Escalation Group is represented by the red line with corresponding standard deviation bars and the Dose Stable Group is represented by the green line with corresponding standard deviation bars. P values at each time point were calculated using a two-way between-group ANOVA using contrasts to evaluate the two groups at each time point and is listed above each time point. B) Tacrolimus dosing is shown over the course of the first month. On the x-axis is the day after kidney transplantation and on the y-axis is the mean tacrolimus dosing of the group (mg/day) corresponding to the day on the x-axis. The Dose Escalation Group is represented by the red line with corresponding standard deviation bars and the Dose Stable Group is represented by the green line with corresponding standard deviation bars. P values at each time point were calculated using a two-way between-group ANOVA using contrasts to evaluate the two groups at each time point and is listed above each time point.

Fig 1 illustrates the tacrolimus trough levels and tacrolimus dosing during the first 28 days of transplantation stratified by the two groups. The tacrolimus trough levels were initially lower in the Dose Escalation Group than in the Dose Stable Group (4.4±2.6 ng/mL vs. 9.7±3.2 ng/mL, respectively) (P<0.001 at day 7, two-way between-group ANOVA using contrasts), but the levels were similar by post-transplant day 28 (7.3±2.2 ng/mL and 9.0±2.1 ng/mL, respectively) (P = 0.22) (Fig 1A). The Dose Escalation Group and the Dose Stable Group had similar initial tacrolimus dosing (4.2±1.1 mg/day vs. 3.8±0.8 mg/day, respectively) (P = 0.61, two-way between-group ANOVA using contrasts), but in response to lower tacrolimus trough levels, the Dose Escalation Group had a higher tacrolimus dosing than the Dose Stable Group by post-transplant day 28 (9.6±2.4 mg/day vs. 3.3±1.5 mg/day, respectively) (P<0.001 at day 28) (Fig 1B).

The Dose Escalation Group had a higher cumulative tacrolimus dosing in the first post- transplantation month than the Dose Stable Group (224±57 mg vs. 101±32 mg, respectively, P = 0.001, Wilcoxon Rank Sum test) and had a lower ratio of tacrolimus trough concentration (ng/mL) to tacrolimus daily dose by weight (mg/kg/day) (C/D) at 1 month post transplantation (a surrogate marker for tacrolimus pharmacokinetics [9, 10]) (66±38 vs. 272±176, respectively, P = 0.003).

The mean±SD initial tacrolimus dosing by weight in the Dose Escalation Group was 0.055±0.026 and 0.052±0.014 mg/kg/day in the Dose Stable Group (P = 0.52, Wilcoxon rank sum test). However, by the end of the first month, tacrolimus dosing by weight was higher in the Dose Escalation Group than in the Dose Stable Group (0.124±0.032 mg/kg/day vs. 0.045±0.023 mg/kg/day, respectively, P = 0.003). Furthermore, within the Dose Stable Group, tacrolimus dosing by weight did not change significantly from initial dosing to 1 month post-transplantation (0.052±0.014 mg/kg/day vs. 0.045±0.023 mg/kg/day, respectively, P = 0.33, Signed Rank test) whereas within the Dose Escalation Group, tacrolimus dosing increased significantly from initial dosing to dosing at 1 month post-transplantation (0.055±0.026 mg/kg/day vs. 0.124±0.032 mg/kg/day, respectively, P = 0.04, Signed Rank test). The Dose Escalation Group and the Dose Stable Group both had a similar number of tacrolimus trough levels measured in the first month of transplantation (8.8±1.9 vs. 8.7±2.1, respectively, P = 0.96).

### Characterization of fecal microbiota in the first month of transplantation

Each of the 19 kidney transplant recipients provided a fecal specimen during the first week of transplantation. We also had 32 additional fecal specimens that were collected after the first specimen and within the first month of transplantation from these 19 kidney transplant recipients. DNA was extracted from all 51 specimens and the V4–V5 region of the 16S rRNA encoding gene was sequenced following PCR amplification. We obtained a total of 1,082,902 sequences from the 51 specimens and we subsampled up to 5000 sequences per sample for subsequent analyses (mean±SD, 4744±808).

**Fig 2:**
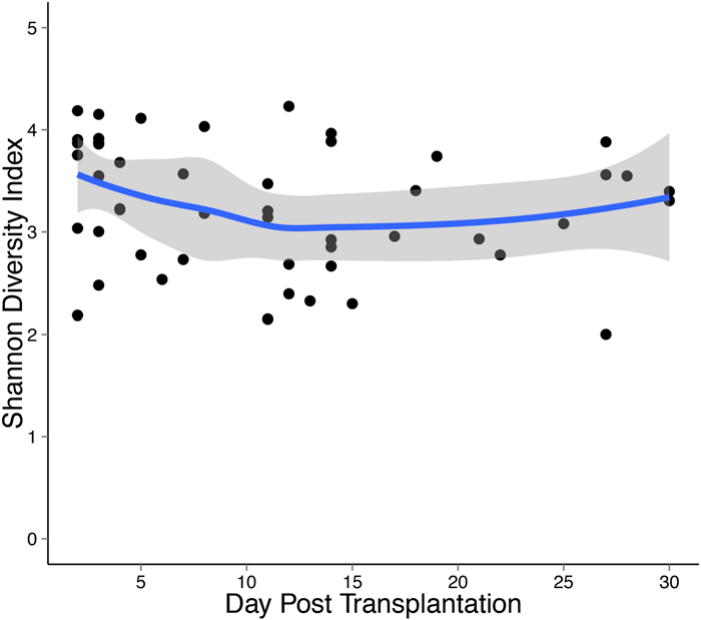
Gut Microbial Diversity During the First Month of Kidney Transplantation. Shannon diversity index is represented over time in all 19 kidney transplant recipients. On the x-axis is the day after kidney transplantation and on the y axis is the Shannon diversity index. Each individual black circle represents a fecal specimen at the time point indicated on the x-axis. The blue line represents a locally estimated scatterplot-smoothed (LOESS) calibration curve with the grey areas representing 95% confidence intervals. As shown, the Shannon diversity index was relatively stable in the study cohort during the first month of transplantation.

In all 51 fecal specimens, we determined the Shannon diversity index which is a combined measure of richness (number of different species) and evenness (their relative abundance in the environment) [35]. The mean (±SD) Shannon diversity index was 3.3±0.6 in the entire group. Fig 2 shows the Shannon diversity index over time in all 19 kidney transplant recipients with a locally estimated scatterplot-smoothed (LOESS) curve. The Shannon diversity index was relatively stable in this cohort of transplant recipients during the first month of transplantation.

**Fig 3:**
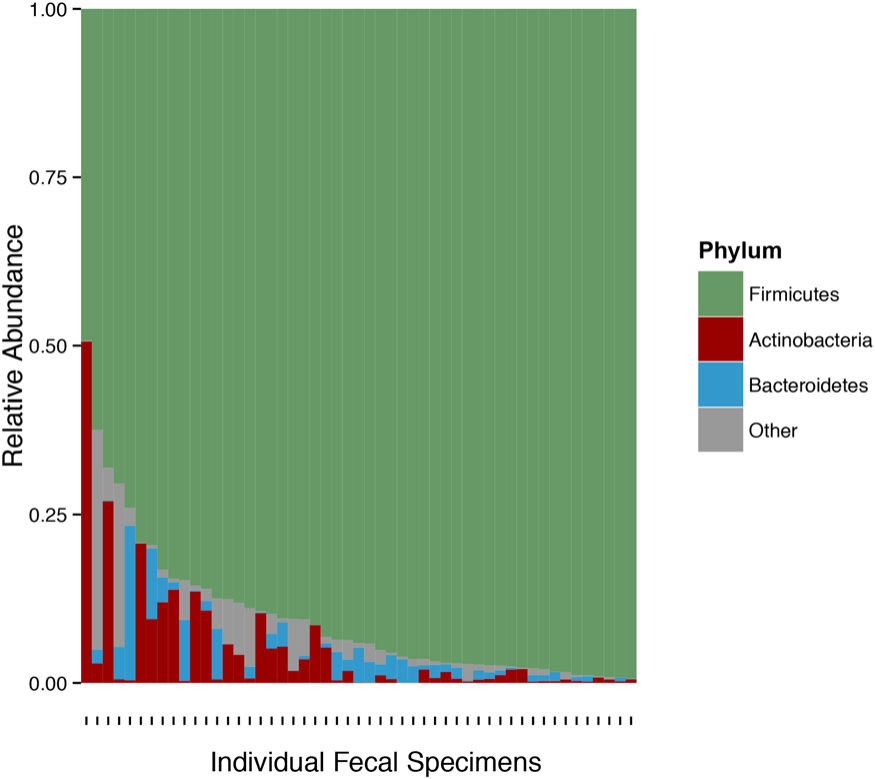
Most Abundant Phyla in the Kidney Transplant Cohort. The relative fecal abundances of the most common phyla are shown for each of the 51 fecal specimens. On the x-axis are individual fecal specimens from the study cohort and on the y-axis is the relative abundance of a taxon. Each bar represents an individual fecal specimen with the color representing the specific phylum indicated in the legend.

In our study cohort, the most common taxa at the phylum level (relative mean abundance >2%) were: Firmicutes (mean 90.5%), Actinobacteria (4.8%), and Bacteroidetes (2.1%) and individual specimens’ relative abundances are shown in Fig 3. At the order level, the most common taxa (>2%) were: Clostridiales (62.2%), Erysipelotrichiales (15.3%), Lactobacillales (10.2%), Bifidobacteriales (4.3%), and Bacteroidales (2.1%). S1 Table lists the most common taxa at the genus (>2%) and the species level (>2%).

### Fecal microbiota early after transplantation and tacrolimus grouping

**Fig 4:**
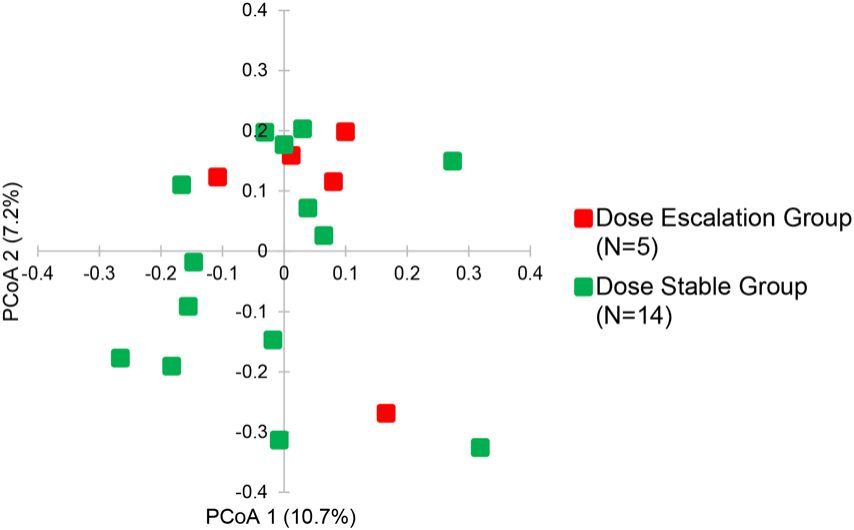
Differences in the Gut Microbiota Between the Tacrolimus Groups. The principal coordinate analyses of the week 1 fecal specimens from the 19 kidney graft recipients are shown. The first two axes of the principal coordinate analysis are represented with principal coordinate axis 1 on the y-axis (10.7% variability) and principal coordinate axis 2 on the x-axis (7.2% variability). The individual red points represent the 5 post-transplantation week 1 fecal specimens from the 5 subjects in the Dose Escalation Group and the individual green points represent the 14 post-transplantation week 1 fecal specimens from the 14 subjects in the Dose Stable Group.

We evaluated whether the fecal microbiota in the first week after kidney transplantation (N = 19) is associated with the tacrolimus dosing groups at one month post- transplantation using principal coordinate analysis. Principal Coordinate Analysis (PCoA) allows for a visualization of dissimilarity between microbiota groups in a 2 dimensional space [34]. Applying PCoA to the unweighted UniFrac distance matrix, fecal specimens in the two dosing groups did not appear to group differently (Fig 4). We also evaluated the Shannon diversity index between the two groups. The mean Shannon diversity index was not significantly different between the Dose Escalation Group and the Dose Stable Group (3.5±0.8 vs. 3.5±0.5, respectively, P = 0.78, Wilcoxon Rank Sum test).

### Fecal *Faecalibacterium prausnitzii* abundance and tacrolimus grouping

In the fecal specimens collected in the first week following kidney transplantation, we compared the most common taxa at the phylum, order, genus, and species levels (taxa comprising >2% within each level) between the Dose Escalation Group and the Dose Stable Group. Table 2 lists the comparisons at each of the taxonomic levels. At the genus level, the week 1 relative abundance of fecal *Faecalibacterium* was significantly higher in the Dose Escalation Group than in the Dose Stable Group (11.8% vs. 0.8%, uncorrected P = 0.002, Wilcoxon rank sum test, P<0.05 after Benjamini-Hochberg correction for multiple hypotheses).

**Table 2:** Comparison of the Most Common Taxa Between the Tacrolimus Groups.

Phylum	Dose Escalation Group (N = 5)	Dose Stable Group (N = 14)	P value (unadjusted)	P value (adjusted)
Firmicutes	0.866	0.906	0.89	0.96
Actinobacteria	0.039	0.036	0.96	0.96
Bacteroidetes	0.016	0.021	0.52	0.96
Order				
Clostridiales	0.611	0.610	0.99	0.99
Erysipelotrichales	0.173	0.165	0.89	0.99
Lactobacillales	0.012	0.087	0.71	0.99
Bifidobacteriales	0.036	0.032	0.71	0.99
Bacteroidales	0.016	0.021	0.52	0.99
Genus				
*Clostridium*	0.103	0.187	0.11	0.55
*Eubacterium*	0.149	0.137	0.82	0.85
*Blautia*	0.065	0.062	0.82	0.85
***Faecalibacterium***	**0.118**	**0.008**	**0.002**	**0.02**
*Streptococcus*	0.010	0.064	0.85	0.85
*Ruminococcus 1*	0.032	0.042	0.56	0.85
*Ruminococcus 2*	0.147	0.085	0.30	0.85
*Bifidobacterium*	0.036	0.032	0.71	0.85
*Coprococcus*	0.025	0.040	0.61	0.85
unclassified *Clostridiales*	0.022	0.034	0.49	0.85
Species				
*Eubacterium dolichum*	0.149	0.121	0.99	0.99
unclassified *Clostridium*	0.057	0.090	0.69	0.99
***Faecalibacterium prausnitzii***	**0.118**	**0.008**	**0.002**	**0.02**
*Blautia producta*	0.016	0.020	0.43	0.91
unclassified *Blautia*	0.016	0.032	0.75	0.99
*Bifidobacterium breve*	0.019	0.009	0.34	0.91
*Streptococcus thermophilus*	0.008	0.025	0.89	0.99
*Ruminococcus bromii*	0.103	0.042	0.14	0.91
*Ruminococcus gnavus*	0.011	0.012	0.96	0.99
unclassified *Coprococcus*	0.020	0.038	0.46	0.91
unclassified *Ruminococcus*	0.043	0.043	0.96	0.99
*Streptococcus lutetiensis*	0.0001	0.030	0.49	0.91
unclassified Clostridiales	0.022	0.034	0.49	0.91

At each of the most common taxa at the phylum, order, genus, and species levels, the mean bacterial abundances in the post-transplantation week 1 fecal specimens from the 5 transplant recipients in the Dose Escalation Group were compared to the mean bacterial abundances in those from the 14 transplant recipients in the Dose Stable Group. Unadjusted p values were calculated using Wilcoxon rank sum tests and the adjusted p values were calculated using the Benjamini-Hochberg correction for multiple hypotheses at each taxonomic level. Fecal *Faecalibacterium prausnitzii* abundance at week 1 post-transplantation was significantly higher in the Dose Escalation Group than in the Dose Stable Group (mean 11.8% vs. 0.8%, respectively, uncorrected P = 0.002, Wilcoxon rank sum test, P<0.05 after Benjamini-Hochberg correction for multiple hypotheses).

**Fig 5:**
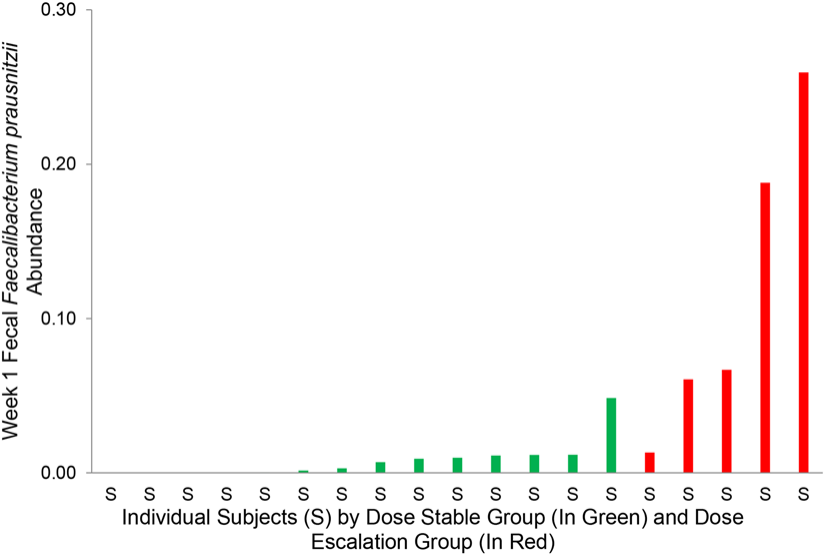
Fecal *Faecalibacterium prausnitzii* Abundance by Tacrolimus Dosing Groups. Each individual subject is represented on the x-axis and the week 1 fecal *Faecalibacterium prausnitzii* abundance is represented on the y-axis. Individual subjects in the Dose Stable Group are represented in green (the 14 subjects on the left) and individuals subjects in the Dose Escalation Group are represented in red (the 5 subjects on the right). The Dose Escalation Group had a significantly higher *Faecalibacterium prausnitzii* proportion than the Dose Stable Group (11.8% vs. 0.8%, respectively, uncorrected P = 0.002, Wilcoxon Rank-Sum test, P<0.05 after Benjamini-Hochberg correction for multiple hypotheses).

Fig 5 illustrates the individual abundance of *Faecalibacterium prausnitzii* in the fecal specimens collected from the 19 recipients in the first week after transplantation by tacrolimus grouping. At the species level, the week 1 relative abundance of fecal *Faecalibacterium prausnitzii* was significantly higher in the Dose Escalation Group than in the Dose Stable Group (11.8% vs. 0.8%, uncorrected P = 0.002, Wilcoxon rank sum test, P<0.05 after Benjamini-Hochberg correction for multiple hypotheses).

There was a continued difference in the fecal *Faecalibacterium prausnitzii* abundance between the two groups in the latter half of the first month of transplantation. Fecal specimens from 14 of the 19 transplant recipients were available for microbial profiling at or after day 14 of transplantation (mean±SD, 23±6 days following transplantation). The mean fecal abundance of *Faecalibacterium prausnitzii* abundance was 20.0% in Dose Escalation Group (N = 5) compared to 5.5% in the Dose Stable Group (N = 9) (P = 0.07, Wilcoxon rank sum test). This difference, however, did not reach the 5% statistical significance.

Fecal specimens collected prior to and following kidney transplantation were available for microbial profiling in 5 of the 19 study subjects. 4 of the 5 subjects belonged to the Dose Stable Group and the remaining 1 subject to the Dose Escalation Group. S1 Fig shows the changes in the abundance of *Faecalibacterium prausnitzii* from pre-transplantation to week 1 post-transplantation in these 5 subjects. Both pre and post-transplantation values were lower in the 4 subjects from the Dose Stable Group compared to the abundance in the one subject from the Dose Escalation Group.

### Correlation between fecal *Faecalibacterium prausnitzii* abundance and tacrolimus dosing

**Fig 6:**
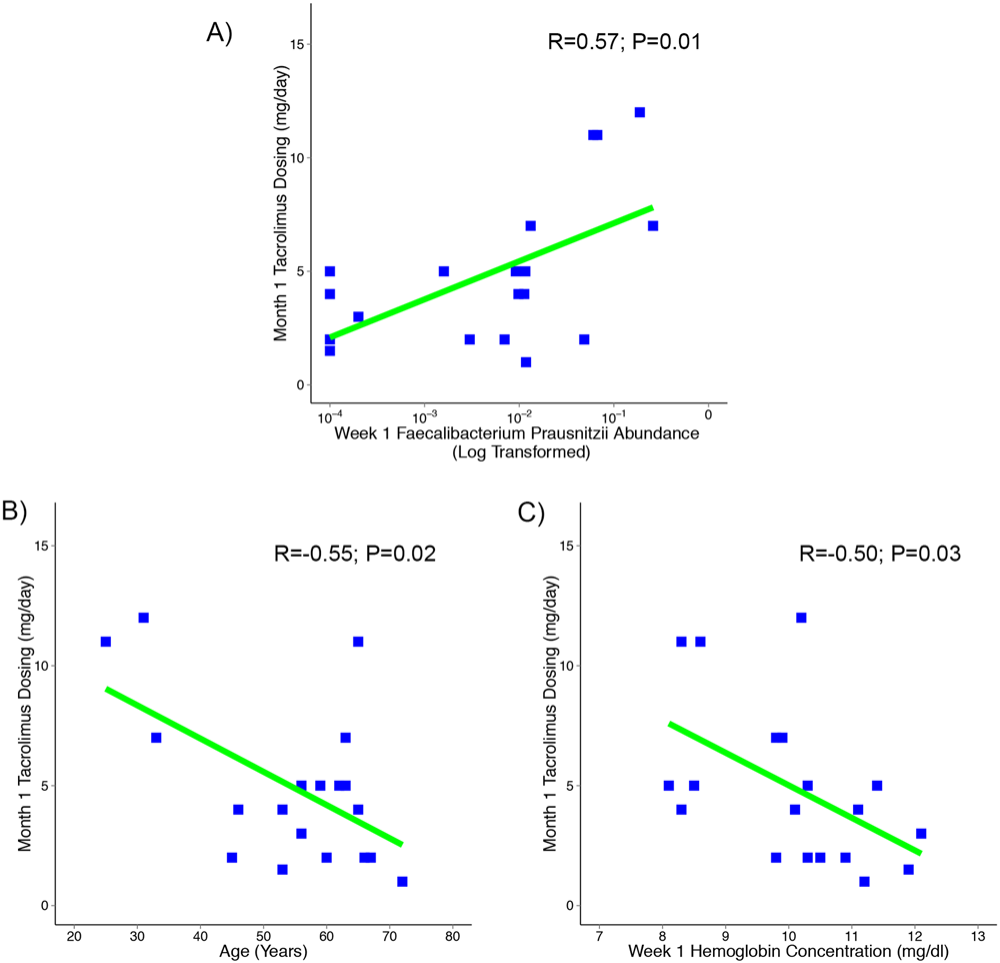
Correlations with Tacrolimus Dosing at 1 Month. A) Fecal *Faecalibacterium prausnitzii* abundance at 1 week post-transplantation is associated with tacrolimus dosing at 1 month. On the x-axis is the log transformed fecal week 1 *Faecalibacterium prausnitzii* abundance and on the y-axis is the corresponding subject’s tacrolimus dosing at 1 month (mg/day). There was a positive correlation between the log-transformed fecal week 1 *Faecalibacterium prausnitzii* abundance and tacrolimus dosing at 1 month (Pearson R = 0.57, P = 0.01). B) Age at transplantation is negatively associated with tacrolimus dosing at 1 month. On the x-axis is the age at transplantation and on the y-axis is the corresponding subject’s tacrolimus dosing at 1 month (mg/day). There was a negative correlation between age and tacrolimus dosing at 1 month (Pearson R = -0.55, P = 0.02). C) Post-transplantation week 1 hemoglobin concentration is negatively associated with tacrolimus dosing at 1 month. On the x-axis is the week 1 hemoglobin concentration and on the y-axis is the corresponding subject’s tacrolimus dosing at 1 month (mg/day). There was a negative correlation between week 1 hemoglobin concentration and tacrolimus dosing at 1 month (Pearson R = -0.50, P = 0.03).

We examined whether post-transplantation week 1 fecal *Faecalibacterium prausnitzii* abundance was associated with the tacrolimus dosing at 1 month. By univariate linear regression, the log transformed *Faecalibacterium prausnitzii* abundance in the fecal specimen collected at 1 week post-transplantation was associated with the 1-month tacrolimus dosing (Coefficent±SE 1.7±0.6, P = 0.01). We also evaluated whether age, transplant recipient’s weight, male gender, African American race, type of transplantation, steroid maintenance protocol, baseline alanine aminotransferase (ALT), baseline albumin concentration, and hemoglobin concentration at one week post- transplantation were correlated with the tacrolimus dosing at one month post-transplantation. Age (Coefficent±SE -0.14±0.05, P = 0.02) and week 1 hemoglobin concentration (Coefficent±SE -1.4±0.6, P = 0.03) were associated with 1-month tacrolimus dosing while weight of the transplant recipient, gender, African American race, type of transplantation (living donor vs. deceased donor), steroid maintenance protocol, baseline ALT, and baseline albumin were not (Table 3). Fig 6 shows the correlation between the log transformed week 1 post-transplantation *Faecalibacterium prausnitzii* abundance and the 1-month tacrolimus dosing (Fig 6A), the correlation between age and the 1-month tacrolimus dosing (Fig 6B), and the correlation between week 1 hemoglobin concentration and the 1-month tacrolimus dosing (Fig 6C). We also examined whether the age of the subject was related to week 1 log-transformed abundance of fecal *Faecalibacterium prausnitzii*. We performed a univariate linear regression between log-transformed abundance of fecal *Faecalibacterium prausnitzii* and subject’s age. This linear regression was not significant (Coefficent±SE -0.03±0.02, P = 0.15).

**Table 3:** Characteristics Associated with Tacrolimus Dosing at 1 Month.

	Univariate Analysis			Multivariable Analysis		
Characteristic	Coefficient	SE	P-value	Coefficient	SE	P-value
Age (years) (continuous)	**-0.14**	**0.05**	**0.02**	**-0.09**	**0.05**	**0.07**
Transplant Weight (kg) (continuous)	0.05	0.06	0.37			
Male Gender (dichotomous)	2.5	1.5	0.11			
African American (dichotomous)	-1.5	2.1	0.49			
Deceased Donor Transplant (dichotomous)	0.14	1.6	0.93			
Steroid Maintenance (dichotomous)	-2.0	1.6	0.21			
Baseline ALT^a^ (IU/L) (continuous)	0.03	0.09	0.77			
Baseline Albumin (mg/dL) (continuous)	0.5	1.5	0.76			
Hgb^b^ 1 Week Post Tx^c^ (mg/dL) (continuous)	**-1.4**	**0.6**	**0.03**	-0.9	0.5	0.10
Log *F*.*Prausnitzii* 1 Week Post Tx^c^ (continuous)	**1.7**	**0.6**	**0.01**	**1.0**	**0.6**	**0.08**

Linear regressions were performed for each of the listed characteristics and the coefficient, standard error (SE), and P values are listed. Characteristics that were associated with tacrolimus dosing at 1 month (P value < 0.10) were computed in a multivariable linear regression and the coefficient, SE, and P values are listed for each of these characteristics.

^a^ ALT: Alanine aminotransferase.

^b^ Hgb: hemoglobin.

^c^ Post Tx: post-transplantation.

We performed a multivariable linear regression with characteristics that were associated with 1-month tacrolimus dosing. The fecal *Faecalibacterium prausnitzii* abundance at week 1 post-transplantation had a Coefficient±SE of 1.0±0.6 (P = 0.08) after controlling for age and week 1 hemoglobin concentration (Adjusted R^2^ value 0.47) (Table 3). This association, however, did not reach 5% statistical significance.

## Discussion

In this pilot study, we characterized the fecal microbiota early after kidney transplantation by 16S rRNA deep sequencing and we report a novel association between fecal *Faecalibacterium prausnitzii* abundance and tacrolimus dosing requirements.

Our characterization of the fecal microbiota after kidney transplantation by deep sequencing is one of the first descriptions of the gut flora early after kidney transplantation. The most commonly observed phyla included Firmicutes, Actinobacteria, and Bacteroidetes, which were also the most common in the Human Microbiome Consortium Project which characterized the fecal bacterial compositions of 242 healthy subjects [36]. Over the course of the first month of transplantation, the diversity of the gut microbiota as measured by the Shannon diversity index was relatively stable in the setting of maintenance immunosuppressive drugs. This is in contrast to the marked decrease in fecal microbiota diversity during the first month of transplantation observed in a series of 94 allogeneic bone marrow transplant recipients [37]. The relative stability in kidney graft recipients may be due to kidney transplant recipients being treated with fewer antibiotics compared to bone marrow transplant recipients.

We identified that the relative fecal abundance of *Faecalibacterium prausnitzii* is associated with tacrolimus dosing in kidney graft recipients. In this study, the relative abundance of *Faecalibacterium prausnitzii* in the first week of transplantation was significantly higher in the Dose Escalation Group than in the Dose Stable Group and was correlated with 1-month tacrolimus dosing.

*Faecalibacterium prausnitzii* is a non-motile gram positive bacterium and is considered one of the most dominant members of the human gut microbial community [38]. It has been reported to produce a substantial amount of butyrate [38, 39]. Butyrate is a major energy source for intestinal cells of the host, is readily absorbed by intestinal epithelial cells, and is implicated in the maintenance of colonic mucosal health [40]. A decrease in *Faecalibacterium prausnitzii* has been associated with inflammatory bowel disease [41]. In this cohort of kidney transplant recipients, fecal *Faecalibacterium prausnitzii* abundance was positively associated with tacrolimus dosing. It is plausible that tacrolimus drug absorption and/or metabolism may be directly linked to a healthy colonic mucosa requiring butyrate from bacterial sources like *Faecalibacterium prausnitzii*. It is also plausible that the colonization of *Faecalibacterium prausnitzii* represents an indirect indicator of a healthy colonic epithelial lining. While tacrolimus is absorbed at the small intestine level, it has also been shown that it can be absorbed at the colonic level [42]. This study, however, did not explore the potential mechanisms through which *Faecalibacterium prausnitzii* may influence tacrolimus metabolism.

Having a functionally healthy intestinal microbiota may impact tacrolimus metabolism through CYP3A4 and P glycoprotein in intestinal epithelial cells and may potentially explain the positive association between *Faecalibacterium prausnitzii* abundance and increased tacrolimus dosing requirements. This may further provide insight into gut disturbances like diarrhea and antibiotic administration. Diarrhea and antibiotic administration have been reported to lead to elevated tacrolimus levels in several studies [17–20, 22–24]. Diarrhea and antibiotic administration are thought to be associated with gut microbial dysbiosis but the types of disturbances have not been characterized at the microbial level. It is possible that fluctuations in bacteria like *Faecalibacterium prausnitzii* may affect tacrolimus metabolism but further studies are needed to explore whether this bacterium or other bacteria that have not been previously identified influence tacrolimus metabolism.

Our study has several limitations. First, this pilot investigation is limited by its small sample size and the statistically significant associations were primarily observed following univariate analyses. Another significant limitation is that tacrolimus dosing is known to be affected by polymorphisms in the CYP3A5 gene [5–9,11] and we did not test for these genetic polymorphisms in our study subjects. Characteristics such as the weight of the transplant recipient, gender, African American race, type of transplantation (living donor vs. deceased donor), corticosteroid use, liver function, and albumin concentration have been associated with tacrolimus dosing [4–11], and our sample size may have been relatively small to appreciate the effects of these variables. It is worth noting that age and the concentration of hemoglobin were negatively associated with tacrolimus dosing in our study, and that there was a trend towards significance that the fecal abundance of *Faecalibacterium prausnitzii* continued to be associated with tacrolimus dosing after controlling for these two variables. Not all transplant recipients in our study had identical induction therapy, preoperative antibiotic use, or *Pneumocystis jiroveci* prophylaxis. However, none of these characteristics were significantly different between the Dose Escalation Group and the Dose Stable Group. Diet can impact gut microbial composition and structure [43] and whether dietary habits contributed to the gut microbial profile observed in this study is not known since we did not systematically collect diet history from our study subjects.

## Conclusions

Our pilot study of adult recipients of kidney allograft identified a novel association between the relative fecal abundance of *Faecalibacterium prausnitzii* and tacrolimus dosing requirements to achieve therapeutic tacrolimus trough levels. This intriguing association between a single bacterial species and the most commonly used calcineurin inhibitor in clinical transplantation gains additional significance in view of tacrolimus’ narrow therapeutic index. Further research may help to provide a more personalized approach to tacrolimus dosing in kidney transplantation as well as to initiate preemptive changes in tacrolimus dosing in the setting of gut microbial dysbiosis from diarrhea or antibiotic administration.

## Supporting Information

S1 Fig Pre- and Post-transplantation Abundance of Fecal *Faecalibacterium prausnitzii*. Five of the 19 subjects had both pre and post fecal specimens available for microbial profiling. The relative abundance of fecal *Faecalibacterium prausnitzii* is shown on the y-axis and the x-axis indicates when the samples were collected (PRETX: pre-transplantation; WEEK 1 POST TX: 1 week post-transplantation). Each line connects an individual subject’s pre-transplantation value to the subject’s post-transplantation value. The 4 subjects from the Dose Stable Group are shown in green and the 1 subject in the Dose Escalation Group is shown in red. Both pre- and post-transplantation values were lower in the Dose Stable Group compared to the Dose Escalation Group.
(PDF)

S1 Information Supporting Information on Clinical and Transplant Characteristics and Fecal Specimens of the Subjects in the Cohort. (XLSX)

S1 Table Most Abundant Taxa in the Gut Microbiota of Kidney Transplant Recipients. For each taxon (>2% relative abundance), the relative mean fecal abundance from the 51 fecal specimens in the cohort is listed at the phylum, order, genus, and species level. The taxa are listed in order of decreasing abundance.
(PDF)
