# Supplementary figures and images for: Gut Microbiota and Tacrolimus Dosing in Kidney Transplantation
Source: PLoS One. 2015 Mar 27;10(3):e0122399. doi: 10.1371/journal.pone.0122399 (PMC4376942; doi:10.1371/journal.pone.0122399)

Figure S1

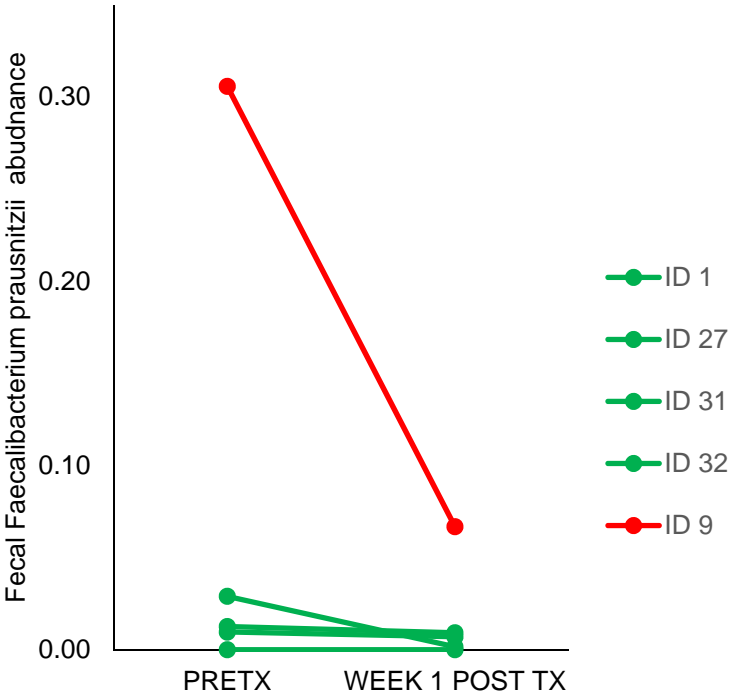

Supplement: S1 Fig — Five of the 19 subjects had both pre and post fecal specimens available for microbial profiling. The relative abundance of fecal Faecalibacterium prausnitzii is shown on the y-axis and the x-axis indicates when the samples were collected (PRETX: pre-transplantation; WEEK 1 POST TX: 1 week post-transplantation). Each line connects an individual subject’s pre-transplantation value to the subject’s post-transplantation value. The 4 subjects from the Dose Stable Group are shown in green and the 1 subject in the Dose Escalation Group is shown in red. Both pre- and post-transplantation values were lower in the Dose Stable Group compared to the Dose Escalation Group. (PDF) [file pone.0122399.s001.pdf]
