# Supplementary material for: Gut Microbiota and Tacrolimus Dosing in Kidney Transplantation
Source: PLoS One. 2015 Mar 27;10(3):e0122399. doi: 10.1371/journal.pone.0122399 (PMC4376942; doi:10.1371/journal.pone.0122399)
Supplement: S1 Table — For each taxon (>2% relative abundance), the relative mean fecal abundance from the 51 fecal specimens in the cohort is listed at the phylum, order, genus, and species level. The taxa are listed in order of decreasing abundance. (PDF) [file pone.0122399.s003.pdf]

Table S1

Phylum	Overall Relative Mean Abundance (%)
Firmicutes	90.5%
Actinobacteria	4.8%
Bacteroidetes	2.1%

Order	Overall Relative Mean Abundance (%)
Clostridiales	62.2%
Erysipelotrichales	15.3%
Lactobacillales	10.2%
Bifidobacteriales	4.3%
Bacteroidales	2.1%

Genus	Overall Relative Mean Abundance (%)
Clostridium	16.5%
Eubacterium	12.6%
Blautia	10.9%
Faecalibacterium	8.2%
Streptococcus	7.5%
Rumminococcus 1	5.2%
Rumminococcus 2	4.8%
Bifidobacterium	4.3%
Coprococcus	2.4%
unclassified *Clostridiales*	2.1%

Species	Overall Relative Mean Abundance (%)
Eubacterium dolichum	11.7%
unclassified *Clostridium*	8.7%
Faecalibacterium prausnitzii	8.2%
Blautia producta	5.3%
unclassified *Blautia*	4.3%
Bifidobacterium breve	3.2%
Streptococcus thermophilus	3.2%

Ruminococcus bromii	3.0%
Ruminococcus gnavus	2.4%
unclassified *Coprococcus*	2.3%
unclassified *Ruminococcus*	2.2%
Streptococcus lutetiensis	2.1%
unclassified Clostridiales	2.1%

At each taxonomic level, the overall relative mean abundance for each taxon from the 51 fecal specimens is listed in order of decreasing abundance.
